# Robust Photocatalytic H_2_O_2_ Production by Octahedral Cd_3_(C_3_N_3_S_3_)_2_ Coordination Polymer under Visible Light

**DOI:** 10.1038/srep16947

**Published:** 2015-11-19

**Authors:** Huaqiang Zhuang, Lifang Yang, Jie Xu, Fuying Li, Zizhong Zhang, Huaxiang Lin, Jinlin Long, Xuxu Wang

**Affiliations:** 1 State Key Laboratory of Photocatalysis on Energy and Environment, College of Chemistry, Fuzhou University, Fuzhou, 350116, P. R. China

## Abstract

Herein, we reported a octahedral Cd_3_(C_3_N_3_S_3_)_2_ coordination polymer as a new noble metal-free photocatalyst for robust photocatalytic H_2_O_2_ production from methanol/water solution. The coordination polymer can give an unprecedented H_2_O_2_ yield of ca. 110.0 mmol • L^−1^ • g^−1^ at pH = 2.8 under visible light illumination. The characterization results clearly revealed that the photocatalytic H_2_O_2_ production proceeds by a pathway of two-electron reduction of O_2_ on the catalyst surface. This work showed the potential perspective of M_x_(C_3_N_3_S_3_)_y_ (M = transitional metals) coordination polymers as a series of new materials for solar energy storage and conversion.

Hydrogen peroxide (H_2_O_2_) is an environmentally benign oxidant widely applied in the areas of organic synthesis, the pulp and paper industry, and disinfection^1^. It is also a block building of post-fossil energy framework as a new solar fuel^2^,^3^,^4^,^5^. However, the traditional anthraquinone method^6^, also referred as the indirect process, for H_2_O_2_ production is contrary to the concept of the modern green chemistry, because it not only involves the multistep reactions of high energy-consuming hydrogenation and oxidation, but also requires large production plants to minimize capital investment and to obtain highly concentrated H_2_O_2_ to reduce transportation costs. The direct synthesis of H_2_O_2_ by the noble metal-catalyzed reaction of molecular oxygen with hydrogen has proven to be feasible^7^,^8^,^9^,^10^,^11^,^12^, but the high production cost and the unsatisfactory efficiency limited its practical application on-large scale. Moreover, some cares have to be required for safe operation because of the potentially explosive danger of H_2_/O_2_ mixture. Recently, the photocatalyzed H_2_O_2_ synthesis has attracted more attention as an “ideal green” technique^13^,^14^,^15^,^16^. Without the presence of external H_2_, H_2_O_2_ can be produced on a micromolar order in O_2_-saturated water by ultraviolet irradiation of TiO_2_^17^. By suppressing the back reaction, the photocatalytic H_2_O_2_ yield can be upgraded to a millimolar level over a surface-fluorinated TiO_2_, but along with a large amount of fluorine contaminant emitted into the aqueous solution^18^. Up to date, much work has devoted to chemically modifying TiO_2_ by noble metal loading or nonmetallic doping for H_2_O_2_ synthesis^19^,^20^,^21^. Carbon nitride (g-C_3_N_4_) and its related composites modified by the electron-deficient aromatic diimide units were recently shown to be also photocatalytically active for H_2_O_2_ synthesis under visible light illumination^22^,^23^,^24^, but it yielded H_2_O_2_ only on no more than a micromolar level. Thus, the development of visible-light-driven photocatalysts effective for safe H_2_O_2_ production on a millimolar and even molar scale remains a formidable challenge.

In natural systems, superoxide dismutases (SODs) are metalloprotein enzymes mildly catalyzed H_2_O_2_ production by the dismutation of superoxide into oxygen and hydrogen peroxide^25^. SODs including three major families of CuZn-SOD, Fe/Mn-SOD and Ni-SOD are essentially coordination compounds with late transition metal ions as central atoms and proteins as ligand. Inspired by these macromolecular metalloproteins, we have long looked for metal coordination polymers as robust photocatalysts to mimic the biocatalytic H_2_O_2_ production. Herein, a C_3_N_3_S_3_-based coordination polymer photocatalyst, Cd_3_(C_3_N_3_S_3_)_2_, was successfully developed to produce H_2_O_2_ on a millimolar level under visible-light irradiation. The yellowish coordination polymer was synthesized by a facile wet-chemical route under the ambient conditions according to the previous work reported by Chudy, J. C. *et al.* who carefully controlled the reaction condition to synthesize the coordination polymer with different stoichiometries^26^. The high stability and low solubility in aqueous solution provided an indicative of the coordination polymer as a catalyst or catalyst support^27^. The elemental analysis of as-synthesed Cd_3_(C_3_N_3_S_3_)_2_ was listed in Table S1, which clearly demonstrates that the molar ratio of C, N, S and Cd elements is 1: 0.98: 0.91: 0.55. The result confirms that the general molecular formula of the resultant product is Cd_3_(C_3_N_3_S_3_)_2_, also denoted as Cd_3_(TMT)_2_ where TMT is 2,4,6-trimercaptotriazine anion. The combination of XRD and FTIR characterizations (Fig. S2,S3, Supporting Information) proves the Cd^2+^-bridged structure of the as-synthesized coordination polymer as depicted in Fig. 1^26^,^27^,^28^.

## Results and Discussion

**Figure 2:**
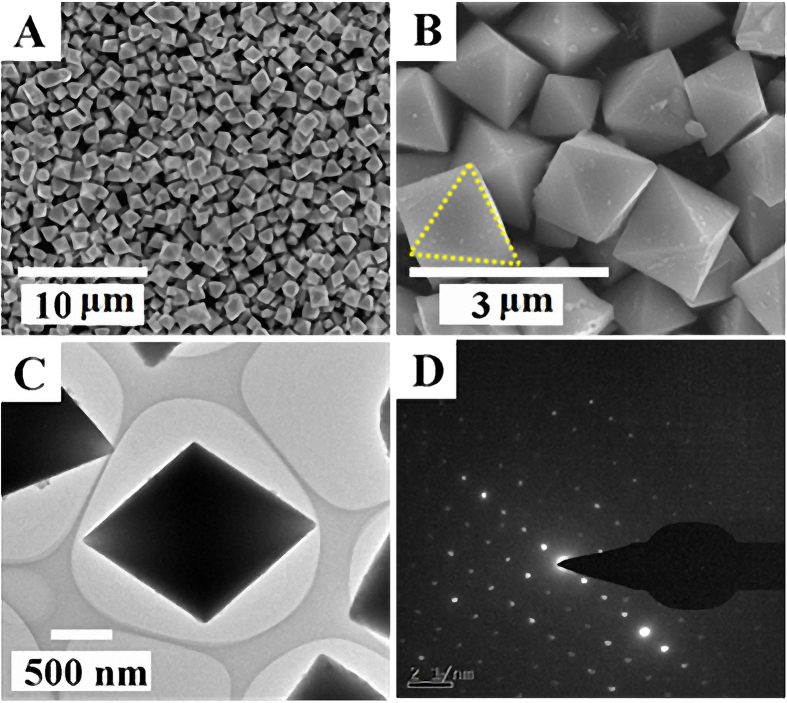
Typical (**A, B**) SEM, (**C**) TEM image and (**D**) the SAED pattern of the Cd_3_(TMT)_2_ coordination polymer.

Figure 2 shows the low-magnification scanning electron microscopy (SEM) (Fig. 2A,B) and transmission electron microscopy (TEM) images (Fig. 2C) of the as-synthesized Cd_3_(TMT)_2_ coordination polymer. It well crystallizes as homogeneously dispersed nanocrystals with the perfect octahedron morphology characteristics. Additionally, it appears that the triangular surface of these octahedrons is sporadically covered by some irregular-shaped nanoparticles, which suggests that the formation of the octahedral nanocrystals maybe follow the “Oriented attachment” mechanism, just similar to the case of a previous work by Zeng and coworkers^29^. Namely, owing to the strong coordination capability of TMT with transition metal ions as well as the low K_sp_ value of Cd_3_(TMT)_2_ in water^27^, the addition of TMT ligand into the Cd^2+^aqueous solution leads first to the formation of Cd_3_(TMT)_2_ nanoparticular precipitate, and long reaction time endows the self-aggregation of these nanoparticles to construct the final 3D architectures. This hypothesis is confirmed by the SEM images of products at different reaction time as shown in Fig. S4 (Supporting Information). The TEM image in Fig. 2C further evidences that the product are structurally well-defined octahedrons with solid inner space. The selected area electron diffraction pattern (SAED) shown in Fig. 2D indicates the single crystal feature of the coordination polymer.

The ultraviolet-visible diffuse reflectance spectrum of the Cd_3_(TMT)_2_ coordination polymer (Fig. S5, Supporting Information) displays a typical optical absorption of semiconductor. The corresponding to the optical band-gap energy of *ca.* 2.76 eV. To further investigate the band structure of the Cd_3_(TMT)_2_ coordination polymer, we also carried out electrochemical analysis. The typical Mott-Schottky plot of Cd_3_(TMT)_2_ in the dark (Fig. S6, Supporting Information) shows a positive slope of C^−2^-E plot, an indicative of n-type semiconductor^30^. The flat-band potential (*V*_fb_) of about −0.78 V *vs.* NHE at pH 7.0 is determined from extrapolation to the *X* intercept in the Mott-Schottky plot. And by combining with the band-gap energy of ca. 2.76 eV estimated from the optical absorption, the valence band position of the Cd_3_(TMT)_2_ coordination polymer is calculated to be 1.98 V *vs.* NHE at pH 7.0. Thus, it is revealed from the band characteristics as illustrated by the insertion in Fig. S6 that, light-excited electrons in the conduction band of the coordination polymer possess a large thermodynamic driving force to reduce O_2_ (*E*°(O_2_/•O_2_^−^) = −0.16 V), and yet the potential of the photogenerated hole in the valence band is inadequate to oxidize OH^-^ to hydroxyl radicals (*E*°(OH^−^/•OH) = 2.4 V). This result clealry indicates that the oxygen reduction reaction over the coordination polymer is feasible.

The activity results of photocatalytic H_2_O_2_ production from methanol aqueous solution confirm the conclusion above. As listed in Table 1, in pure water (Entry 1), the Cd_3_(TMT)_2_ semiconductor is reluctant to produce H_2_O_2_ under the indicated conditions. On the contrary, with the addition of methanol as hole scavenger and proton donor, which is beneficial for the separation of electron-hole pairs, 1.5 mmol•L^−1^ H_2_O_2_ is produced under visible-light irradiation (Entry 2). Notably, this yield is highly comparable to the system using Au-Ag/TiO_2_ under ultraviolet light irradiation as previously reported^20^. But in dark (Entry 3), no H_2_O_2_ is produced, confirming that the H_2_O_2_ production is driven by light absorption. According to the redox potentials of the electron/hole pairs of the coordination polymer, H_2_O_2_ can be stoichiometrically formed in an aerated aqueous solution via two different pathways as follows:


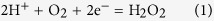



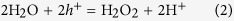


Our control experiments uncover some basic mechanism of the Cd_3_(TMT)_2_-catalyzed H_2_O_2_ evolution. When the system is bubbled with N_2_ to eliminate O_2_, no H_2_O_2_ is detected with KMnO_4_ titration method, indicating the participation of O_2_ in the photocatalytic H_2_O_2_ production. Thus, it can be concluded that the second potential pathway does not contribute to the H_2_O_2_ production in our system. This conclusion is also confirmed by our further control experiments (Entries 5–7). If water oxidation, as described by equation (2), is primarily responsible for the photocatalytic H_2_O_2_ production, and then increasing the concentration of H^+^will deteriorate the photocatalytic H_2_O_2_ production of the Cd_3_(TMT)_2_ semiconductor. However, it is shown that, the H_2_O_2_ concentration increases with the H^+^concentration in the solution, and the concentration of H_2_O_2_ reaches to about 8.75 mmol•L^−1^ at pH = 2.8. In addition, when AgNO_3_, which is an often-used electron trapper^31^, was added in the system (Entry 8), no detectable H_2_O_2_ is produced in the solution after 4 h of visible-light irradiation. It indicates the pivotal role of photo-electrons for H_2_O_2_ generation. Based on the activity results of the control experiments we can confirm that, in the case of Cd_3_(TMT)_2_-photocatalyzed H_2_O_2_ production, the overall reaction would be described by the equation (1), which features two-electron reduction of O_2_. It is also noteworthy that, in most cases as previously reported, the produced H_2_O_2_, a more reactive oxidation agent than O_2_, is presumably quick rebound and readily suffers from the reduction reaction by photo-generated electrons on the catalyst, which results in a very low efficiency of H_2_O_2_ evolution^21^. Interestingly, in the present study, the accumulation of H_2_O_2_ with concentration up to several millimoles per liter is achieved. It should be ascribed to the rapid desorption of H_2_O_2_ from the surface of the Cd_3_(TMT)_2_ photocatalyst, which suppresses the photocatalytic H_2_O_2_ decomposition.

**Figure 3:**
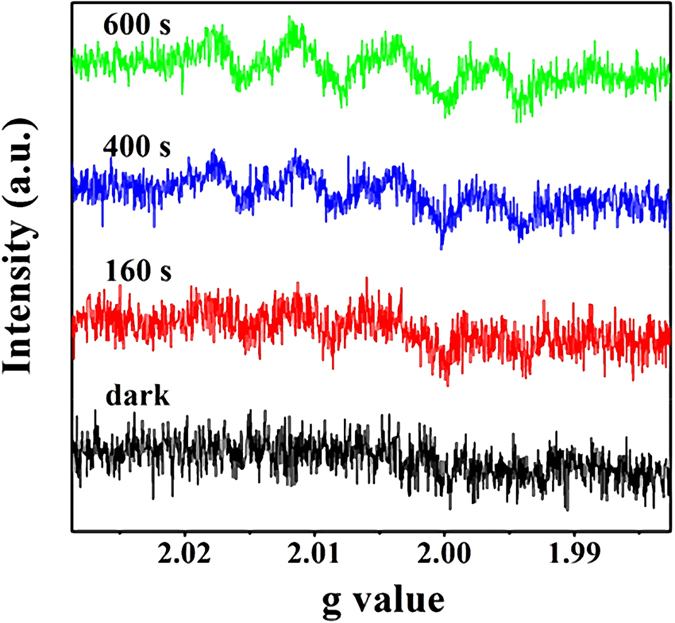
ESR spectra of DMPO–•O_2_^−^/•OOH adduct in the Cd_3_(TMT)_2_/DMPO system before and after visible light irradiation.

5,5-dimethyl-1-pyrroline N-oxide (DMPO) trapping electron paramagnetic resonance (ESR) analysis was used to identify the intermediate oxygen species formed during the H_2_O_2_ evolution, as shown in Fig. 3. No ESR signal can be observed in dark. On the contrary, upon visible light irradiation (λ ≥ 420 nm), a set of ESR signals of DMPO-•O_2_^−^/•OOH adduct (•O_2_^−^, a product derived from oxygen reduction reaction: O_2_ + *e*^−^ = •O_2_^−^) is discernable within 160 s, and no ESR signals of DMPO−•OH adduct occurs (Fig. S7, Supporting Information), indicating the absence of H_2_O_2_ decomposition induced by reduction reaction with electrons (*e.g.* H_2_O_2_ + e^−^ = OH^-^ + •OH)^32^. Furthermore, the intensity of the DMPO-•O_2_^−^/•OOH adduct signals increase gradually with irradiation time. All these facts are in good agreement with the activity results, indicating that the H_2_O_2_ evolution over the Cd_3_(TMT)_2_ coordination polymer proceeds *via* the O_2_ reduction process, as illustrated by equation (1).

**Figure 4:**
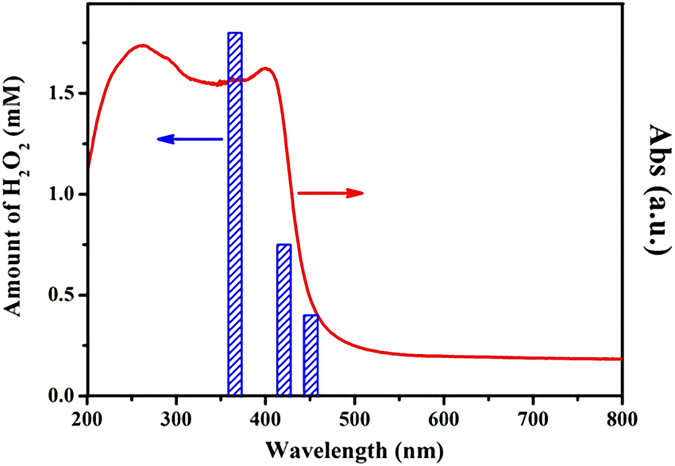
Wavelength-dependent hydrogen peroxide evolution by Cd_3_(TMT)_2_ coordination polymer. Reaction conditions: 80 mg catalyst dispersed in 19 ml distilled water mixed with 1 ml methanol, room temperature. The reaction time is 4 hours.

**Figure 5:**
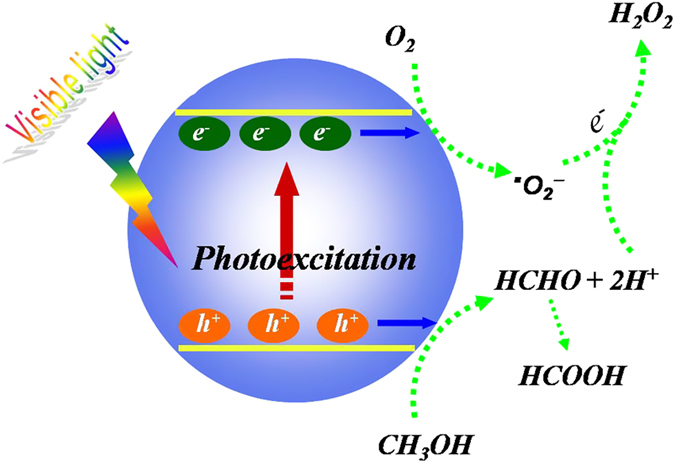
Proposed mechanism of the H_2_O_2_ production on the visible-light-activated Cd_3_(TMT)_2_ under the ambient condition.

The spectrum action of H_2_O_2_ production as shown in Fig. 4 further validates that the photoreaction proceeds through light-excitation of the coordination polymer. It appears that the H_2_O_2_ amount produced in the system decreases with increasing the incident light wavelength, matching well with the optical spectrum. This result clearly indicates that the H_2_O_2_ production is intrinsically a photocatalytic process driven by photoexcitation of the coordination polymer semiconductor. Therefore, we can propose a reasonable mechanism of Cd_3_(TMT)_2_-photocatalyzed oxygen activation for H_2_O_2_ production, as illustrated in Fig. 5. Under visible light irradiation, the electron-hole pairs are produced, and then methanol is oxidized by holes into formaldehyde and proton (eq. 3), which contributes to the separation of charge carriers and H^+^^33^,^34^,^35^. The adsorbed oxygen molecules are spontaneously reduced by electrons to form superoxide radicals (eq. 4), which further react with protons to produce •OH_2_ radicals (eq. 5). The •OH_2_ radicals can readily undergo further reduction with e^-^ (eq. 6), producing HO_2_^-^ anions. Finally, just as demonstrated by equation (7), the negatively-charged HO_2_^-^ reacts with H^+^, leading to the evolution of the final H_2_O_2_ product^36^.


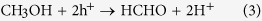



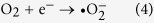



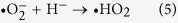



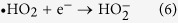



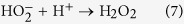


**Figure 6:**
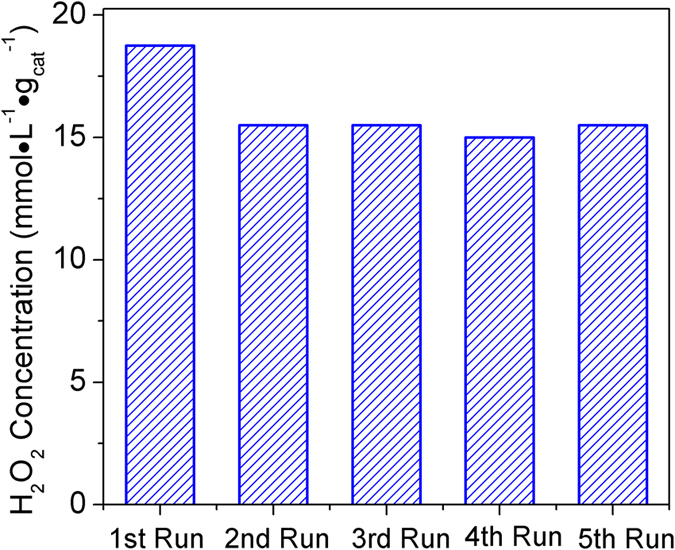
Stability testing of photocatalytic activity of the Cd_3_(TMT)_2_ coordination polymer.

In order to clearly verify the photocatalytic reaction mechanism, the concentration of formic acid and formaldehyde has been further measured by the ion chromatography and acetylacetone spectrophotometry, as shown in Fig. S8. The concentration of HCHO and HCOOH gradually increases with the enhancement of reaction time under 24 h of visible-light irradiation, which is good consistent with the above reaction mechanism. Although the accumulated H_2_O_2_ can further oxidize HCHO into HCOOH, which will lead to some H_2_O_2_ loss, a stable H_2_O_2_ concentration in the aqueous solution can be achieved once a production-decomposition balance of H_2_O_2_ is reached, just as demonstrated by Fig. S9 (Supporting Information) representing a time curve of enzyme-biocatalyzed H_2_O_2_ evolution, over the coordination polymer from methanol aqueous solution. As we all known, the formation and decomposition of H_2_O_2_ follow zero- and first-kinetics toward H_2_O_2_ concentration, respectively^14^,^20^. Therefore, the kinetic data can be modeled and explained by the equation: [H_2_O_2_] = (k_f_/k_d_){1-exp(1-k_d_t)}, where t is time, k_f_ (mM h^−1^) and k_d_ (h^−1^) are the formation and decomposition rate constants for H_2_O_2_, respectively. The k_f_ and k_d_ values of Cd_3_(TMT)_2_ polymer are 0.39 mM h^−1^and 0.04 h^−1^, respectively, indicating that the Cd_3_(TMT)_2_ polymer is a robust photocatalyst for H_2_O_2_ production. To check the photo-stability of the as-prepared photocatalyst, the photocatalytic evolution of H_2_O_2_ was repeated up to five cycles under the same conditions (Fig. 6). It can be clearly seen that after five successive operations, the coordination polymer still maintains the high photocatalytic activity for H_2_O_2_ production. In addition, its crystal structure does not change after photocatalytic reaction, as shown in Fig. S10 (Supporting Information). Those results indicate that the Cd_3_(TMT)_2_ polymer is able to serve as a stable, reusable photocatalyst for H_2_O_2_ generation from methanol/water solution.

According to the characterization results above, we believe that the Cd_3_(TMT)_2_ coordination polymer can fulfill as a versatile visible-light photocatalyst. The activity results of photocatalytic degradation of Rhodamine B over Cd_3_(TMT)_2_ shown in Fig. S11 (Supporting Information) also confirms that it indeed enables the destruction of organic pollutants due to the •O_2_^−^ generation. Importantly, a considerable amount of H_2_O_2_ is simultaneously produced along with the Rhodamine B photodegradation in the solution^37^ (Fig. S12, Supporting Information). This result suggests that the sacrificial agent, methanol, will be hopefully replaced by waste organic dyes for H_2_O_2_ production in the future, synchronously achievement of environmental remediation.

In summary, a bioinspired metal coordination polymer with a general molecular formula of Cd_3_(TMT)_2_ was reported for the first time to fulfill as a visible light photocatalyst effective for H_2_O_2_ evolution with the aid of methanol. The coordination polymer features a well-defined octahedral morphology and high crystallinity and shows robust photocatalytic H_2_O_2_ production on a millimolar level. The electrochemical analysis and ESR characterizations clearly reveal that the photocatalytic H_2_O_2_ evolution over the coordination polymer follows a mechanism of two-electron reduction of O_2_. This work shows the potential promising of the transitional metal coordination polymers in solar energy storage and conversion, especially organic photosynthesis.

## Methods

### Materials

Cd(NO_3_)_2_ • 4H_2_O and sodium hydroxide (NaOH) were supplied by Sinopharm chemical reagent Co., Ltd (Shanghai, China), trithiocyanuric acid (H_3_TMT) was purchased from Tokyo Chemical Industry Co., Ltd (Tokyo, Japan). All materials are analytical grade purity without further purification prior to use. Deionized (DI) water used in the synthesis was obtained from local sources.

### Catalyst Preparation

The monodisperse Cd_3_(TMT)_2_ octahedrons are prepared by a facile template-free wet-chemical synthesis at room temperature. Typically, 0.015 mol cadmium nitrate, Cd(NO_3_)_2_ • 4H_2_O, was dissolved in 200 mL DI water under mechanically stirring to form a transparent solution (denoted as solution A). Trithiocyanuric acid (0.01 mol) was dissolved in 200 mL 0.15 mol • L^−1^ NaOH aqueous solution to form yellowish homogenous solution, which is denoted as solution B. In order to avoid the formation of cadmium hydroxide precipitation, solution B was slowly added into solution A drop-by-drop under vigorously stirring. Afterwards, the system was aged for 24 h with mildly stirring. The products were then separated by filtration, washed by DI water, and fully dried at 333 K in oven to get the final resultants, namely Cd_3_(TMT)_2_ octahedrons.

### Characterizations

The phase composition of the as-prepared samples was determined on a Bruker D8 Advance X-ray diffractometer (XRD) using Ni-filtered Cu Kα radiation at 40 kV and 40 mA in the 2*θ* ranging from 20° to 80° with a scan rate of 0.02° per second. Field-emission scanning electron microscopy (FE-SEM) was used to characterize the morphology and elemental distribution of the as-prepared samples on a FEI Nova NANOSEM 230 spectrophotometer. Transmission electron microscopy (TEM), high-resolution transmission electron microscopy (HRTEM) images and energy-dispersive X-ray spectroscopy (EDX) were obtained using a JEOL model JEM 2010 EX instrument at an accelerating voltage of 200 kV. The optical properties of the as-prepared samples were analyzed by UV–vis diffuse reflectance spectroscopy (DRS) using a UV–vis spectrophotometer (Cary 500, Varian Co.), in which BaSO_4_ was employed as the internal reflectance standard. X-ray photoelectron spectroscopy (XPS) measurement was carried out on a Thermo Scientific ESCA Lab 250 spectrometer which consists of a monochromatic Al Kα as the X-ray source, a hemispherical analyzer and sample stage with multi-axial adjustability to obtain the surface composition of the sample. All of the binding energies were calibrated by the C 1 s peak at 284.6 eV. The concentration of formic acid was measured by the ion chromatography (Dionex, ICS-1100). Electron spin resonance (ESR) signal of the radicals spin-trapped by 5,5-dimethyl-l-pyrroline-N-oxide (DMPO) was recorded on a Bruker EPR A300 spectrometer. The irradiation source (λ ≥ 420 nm) was a 300 W Xe arc lamp system and the whole ESR experiment was measured under room temperature. The settings for the ESR spectrometer were as follows: center field = 3507 G, microwave frequency = 9.84 GHz and power = 6.36 mW. The Mott-Schottky experiments were obtained on a Precision PARC workstation. The electrochemical analysis was carried out in a conventional three-electrode cell using a Pt plate and an Ag/AgCl electrode as the counter electrode and reference electrode, respectively. For electrode preparation, indium-tin oxide (ITO) glass was firstly cleaned by sonication in ethanol for 30 min and dried at 353 K. The boundary of ITO glass was protected using scotch tape. 5 mg of sample was dispersed in 0.5 mL ethanol by sonication to get a slurry. The slurry was spread onto the pretreated ITO glass. After air drying, the working electrode was further dried at 393 K for 2 h to improve adhesion. Then the scotch tape was unstuck and the uncoated part of the electrode was isolated with epoxy resin. The exposed area of the working electrode was 0.25 cm^2^. Mott-Schottky experiments were measured in a sodium sulfate electrolyte solution (0.2 M) (pH = 6.8), the potential ranged from −0.2 V to 0.8 V, and the perturbation signal were 10 mV with the frequency at 1 K Hz.

### Photocatalytic activity test

In a typical photocatalytic reaction, a 300 W Xe arc lamp (PLS-SXE 300, Beijing Perfectlight Co., Ltd.) with a UV−CUT filter to cut off light of wavelength <420 nm was used as the irradiation source. 80 mg of photocatalyst was added into 20 mL of the methanol aqueous solution (19 mL H_2_O with 1 mL methanol). Before visible light illumination, the above suspension was stirred in the dark for 1 h to ensure the establishment of adsorption-desorption equilibrium between the sample and reactant. During the process of the reaction, 5 mL of sample solution was collected after 4 hours of visible light irradiation and centrifuged to remove the catalyst completely at 12000 rpm. Afterward, the concentration of H_2_O_2_ was determined by KMnO_4_ titration (*c*_KMnO4_ = 1 mmol • L^−1^) with the addition of 5 mL 1 M H_2_SO_4_ solution. When the solution becomes the pink after the addition of KMnO_4_ solution and keeps the color of solution for 30 s, the concentration of KMnO_4_ solution is equivalent to the concentration of H_2_O_2_. All of the experimental processes were conducted under ambient temperature.

## Additional Information

**How to cite this article**: Zhuang, H. *et al.* Robust Photocatalytic H_2_O_2_ Production by Octahedral Cd_3_(C_3_N_3_S_3_)_2_ Coordination Polymer under Visible Light. *Sci. Rep.* **5**, 16947; doi: 10.1038/srep16947 (2015).

## Supplementary Material

Supporting Information

## Figures and Tables

**Figure 1:**
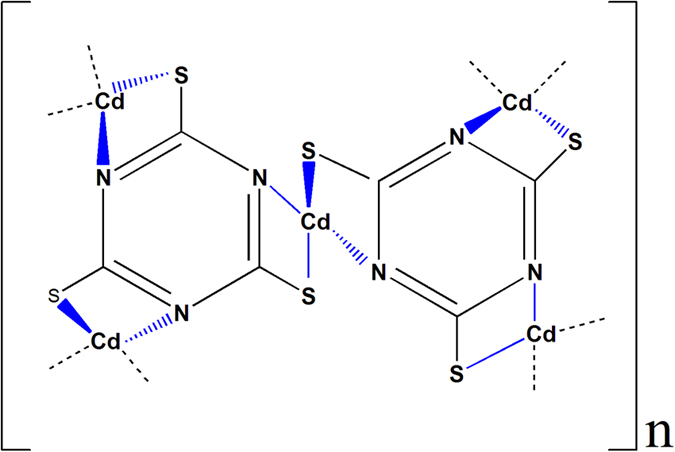
Structure of the Cd_3_(TMT)_2_ coordination polymer.

**Table 1:** Photocatalytic H_2_O_2_ evolution over Cd_3_(TMT)_2_ under different conditions.

Entry	pH	hν	Atmosphere	C(H_2_O_2_) [mmol • L^−1^]
1^[a]^	6.7	+	air	negligible
2^[b]^	6.7	+	air	1.5
3^[b]^	6.7	−	air	negligible
4^[b]^	6.7	+	N_2_	negligible
5^[c]^	5.8	+	air	1.75
6^[c]^	4.1	+	air	2.0
7^[c]^	2.8	+	air	8.75
8^[d]^	6.7	+	air	negligible

[a] Reaction conditions: 80 mg catalyst dispersed in 20 ml distilled water, visible light (λ ≥ 420 nm), room temperature, the reaction time is 4 hours; [b] Reaction conditions: 80 mg catalyst dispersed in 19 ml distilled water mixed with 1 ml methanol, other conditions as [a]; [c] pH of the solution was adjusted by *con*. HNO_3_, other conditions as [b]. [d] With the addition of AgNO_3_ as electron trapper, other conditions as [b]. The concentration of produced H_2_O_2_ was determined by KMnO_4_ titration^20^.
