# Supplementary material for: Robust Photocatalytic H2O2 Production by Octahedral Cd3(C3N3S3)2 Coordination Polymer under Visible Light
Source: Sci Rep. 2015 Nov 19;5:16947. doi: 10.1038/srep16947 (PMC4652267; doi:10.1038/srep16947)
Supplement: Supporting Information [file srep16947-s1.doc]

**Robust Photocatalytic H_2_O_2_ Production by Octahedral Cd_3_(C_3_N_3_S_3_)_2_ Coordination Polymer under Visible Light**

Huaqiang Zhuang, Lifang Yang, Jie Xu, Fuying Li, Zizhong Zhang, Huaxiang Lin, Jinlin Long*, Xuxu Wang

State Key Laboratory of Photocatalysis on Energy and Environment, College of Chemistry, Fuzhou University, Fuzhou, 350116, P.R. China

*E-mail Address: [jllong@fzu.edu.cn](mailto:jllong@fzu.edu.cn)

Tel: +86-591-83779121; fax: +86-591-83779251

**Table S1 Elemental Analysis of the coordination polymer**

| **Elements** | **C** | **N** | **S** | **Cd** |
| --- | --- | --- | --- | --- |
| **Contents (%)** | **10.28** | **11.71** | **24.92** | **53.57** |
| **Molar ratio** | **C:N:S:Cd = 1: 0.98: 0.91: 0.55** | | | |

**
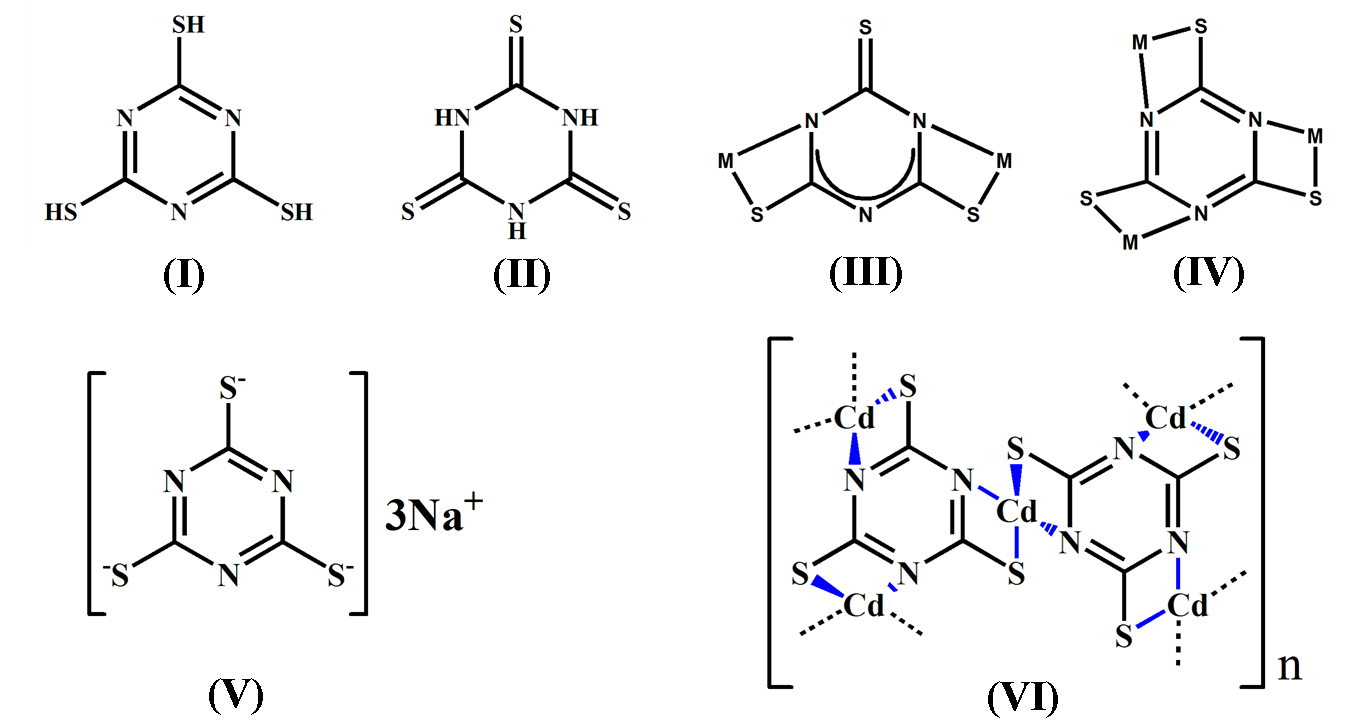
**

**Figure S1** Summary of different structural formulas


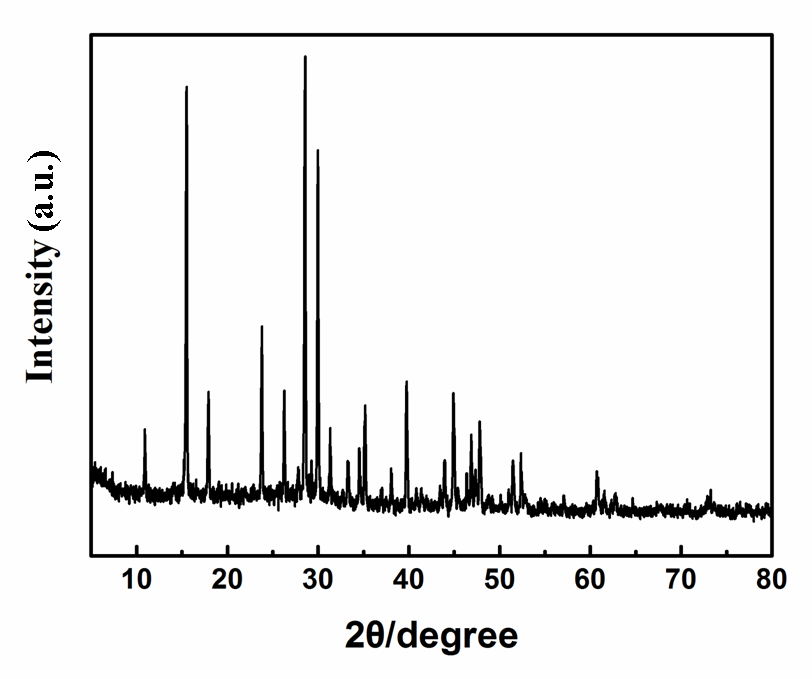


**Figure S2** XRD pattern of the as-synthesized Cd_3_(TMT)_2_.

**
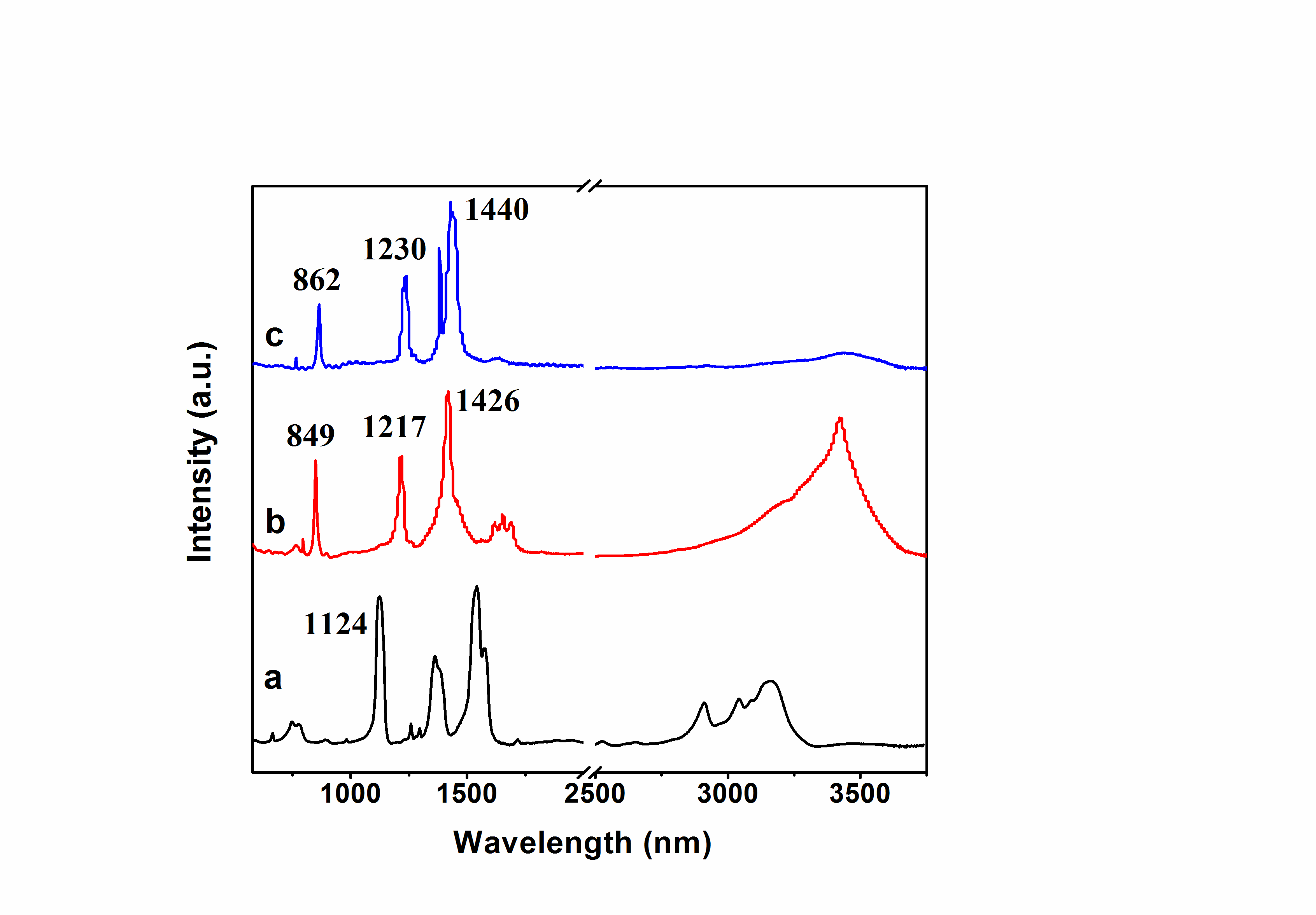
**

**Figure S3** FTIR spectra of H_3_TMT (a), Na_3_TMT (b) and Cd_3_(TMT)_2_ (c).

The XRD pattern of the as-synthesized Cd_3_(TMT)_2_ is shown in the **Fig. S2**. The strong diffraction peaks demonstrate a better crystallinity for Cd_3_(TMT)_2_ sample and the main diffraction peaks are good agreement with the previous work reported by Atwood *et al*^1^, suggesting that the Cd_3_(TMT)_2_ sample with high crystallinity was successfully synthesized in the present study.

The IR spectrum of trithiocyanuric acid (H_3_TMT) is well consistent with that reported in literatures.[^2^](#_ENREF_1) The major bands at 1540, 1124, and 750 cm^-1^ in the IR spectrum are characteristic of the nonaromatic, thirthione form of the TMT ring system.^3,4^ Specifically, the 1124 cm^-1^ band is assigned to C=S stretching vibrations. The peaks at the position of 2900 – 3160 cm^-1^ are attributed to -N-H stretching vibrations. This indicates that, in the solid state, H_3_TMT exists in the non-aromatic trithione, as depicted in **Fig. S1**(II), rather than in the aromatic trithiol (**Fig. S1**(I)) form.

It is demonstrated that there may be two possible structures of metal-TMT complex, as displayed by **Fig. S1**(III) and **Fig. S1**(IV).[^5^](#_ENREF_1) In order to study the structure of the as-synthesized Cd_3_(TMT)_2_ compound, we first examined the IR spectrum of 2,4,6-trimercaptotriazine trisodium salt (Na_3_TMT), as shown in **Fig. S3(b)**. Since from earlier report, the structure of Na_3_TMT is unambiguous known, as demonstrated in **Fig. S1**(V), in which TMT moiety exists in the aromatic form.[^6^](#_ENREF_2) Thus, the three major peaks observed that locate at 1426, 1217 and 849 cm^-1^, respectively, can be regarded as the characteristic of the aromatic trithiol form of the TMT ring system. **Fig. S3(c)** displays the IR spectrum of as-prepared Cd_3_(TMT)_2_ sample. As can be clearly seen, the three characteristic bands shift positively together, signifying that a complex has formed. Furthermore, no band originating from C=S stretching vibrations can be found, which excludes the possible structure of the coordination compound as displayed by **Fig. S1**(III).

Based on the above analysis in combination with the elemental analysis which demonstrated that the metal/ligand ratio in the coordination compound is 1.5, the most plausible structure of Cd_3_(TMT)_2_ can be illustrated by **Fig. S1**(VI), which is in coincidence with the previous report by Chudy *et al*.[^7^](#_ENREF_1)


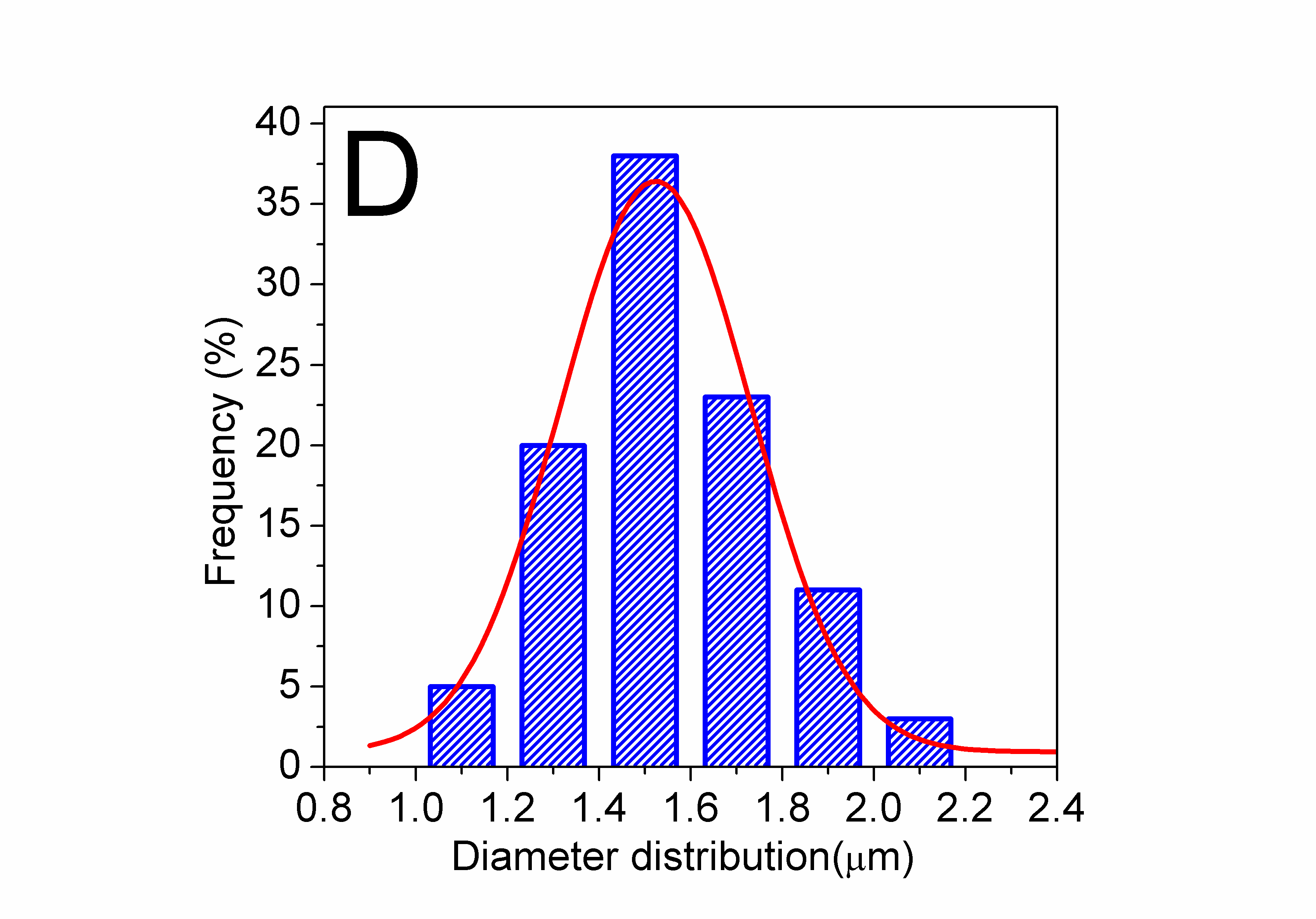

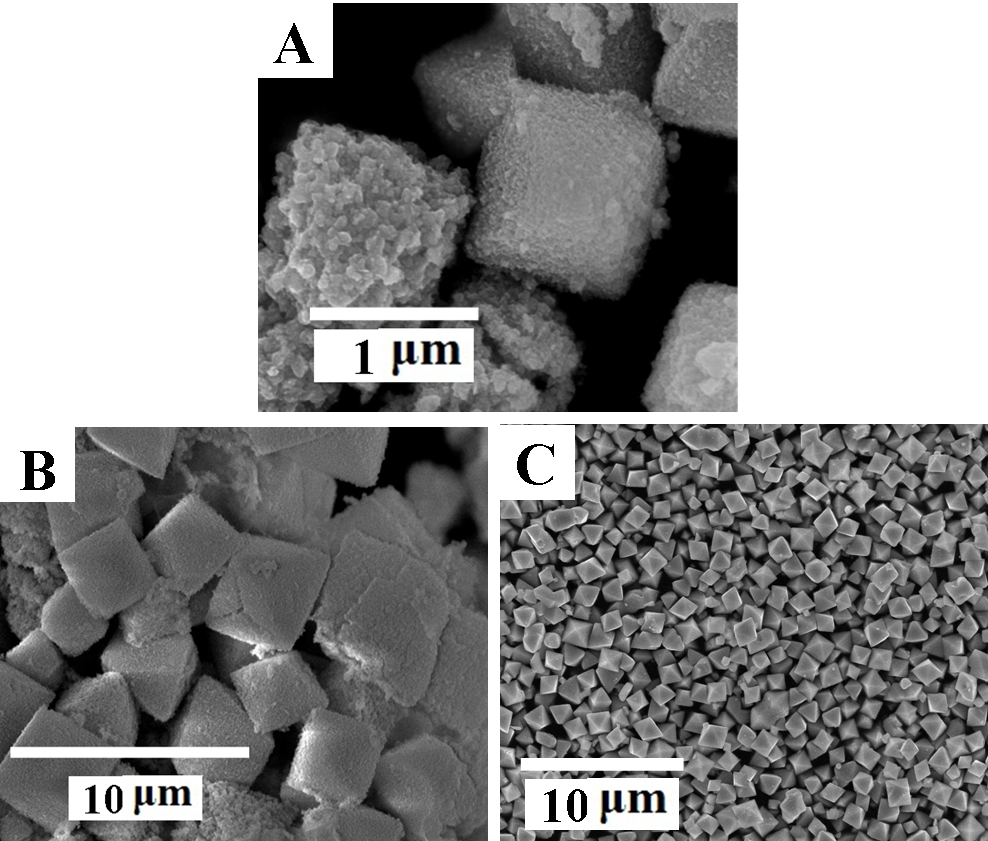

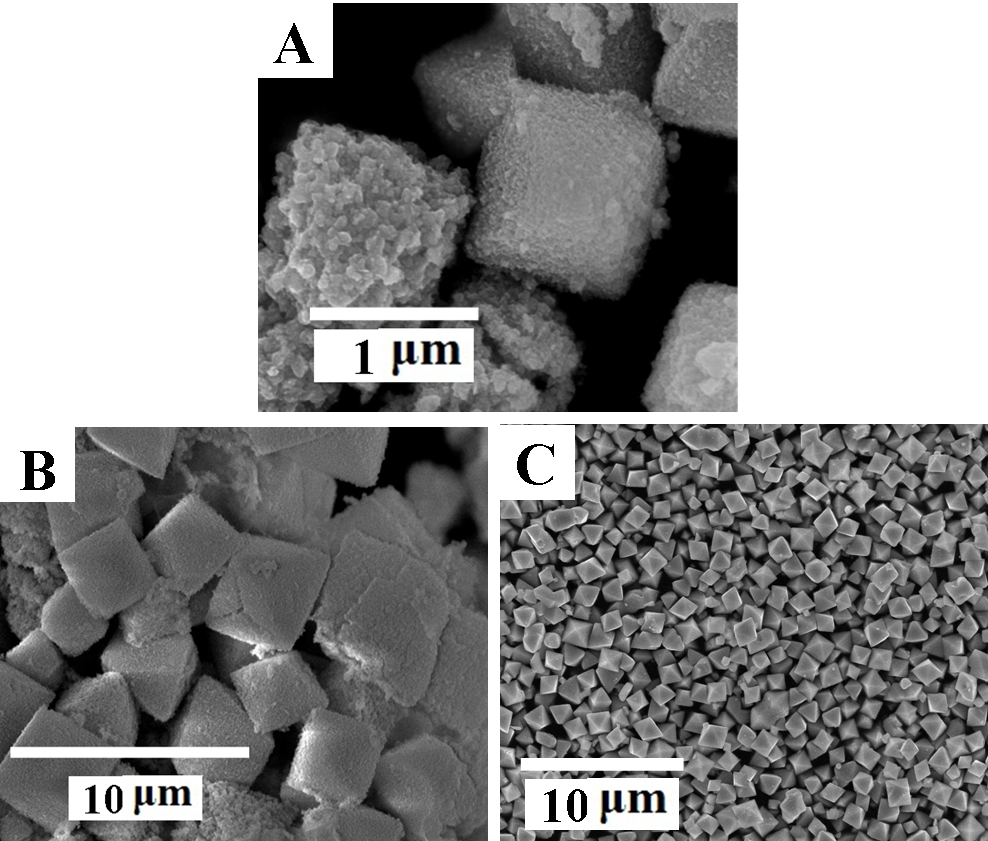

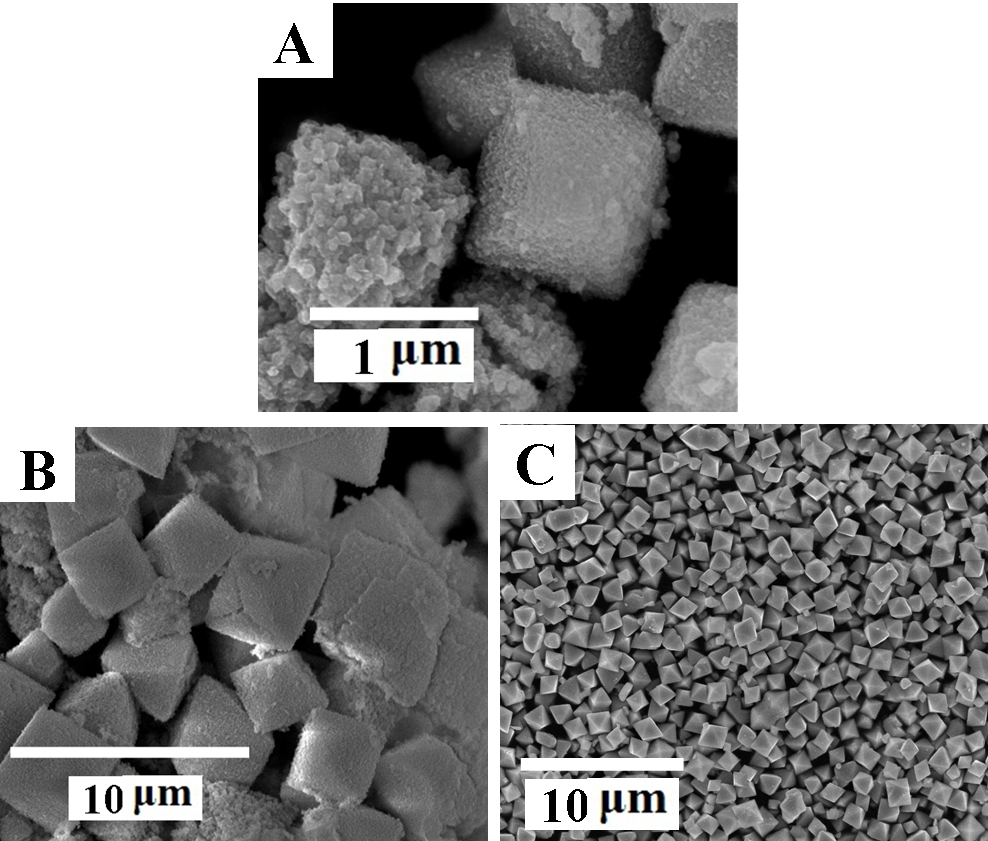


**Figure S4** Morphology evolution during the preparation process.


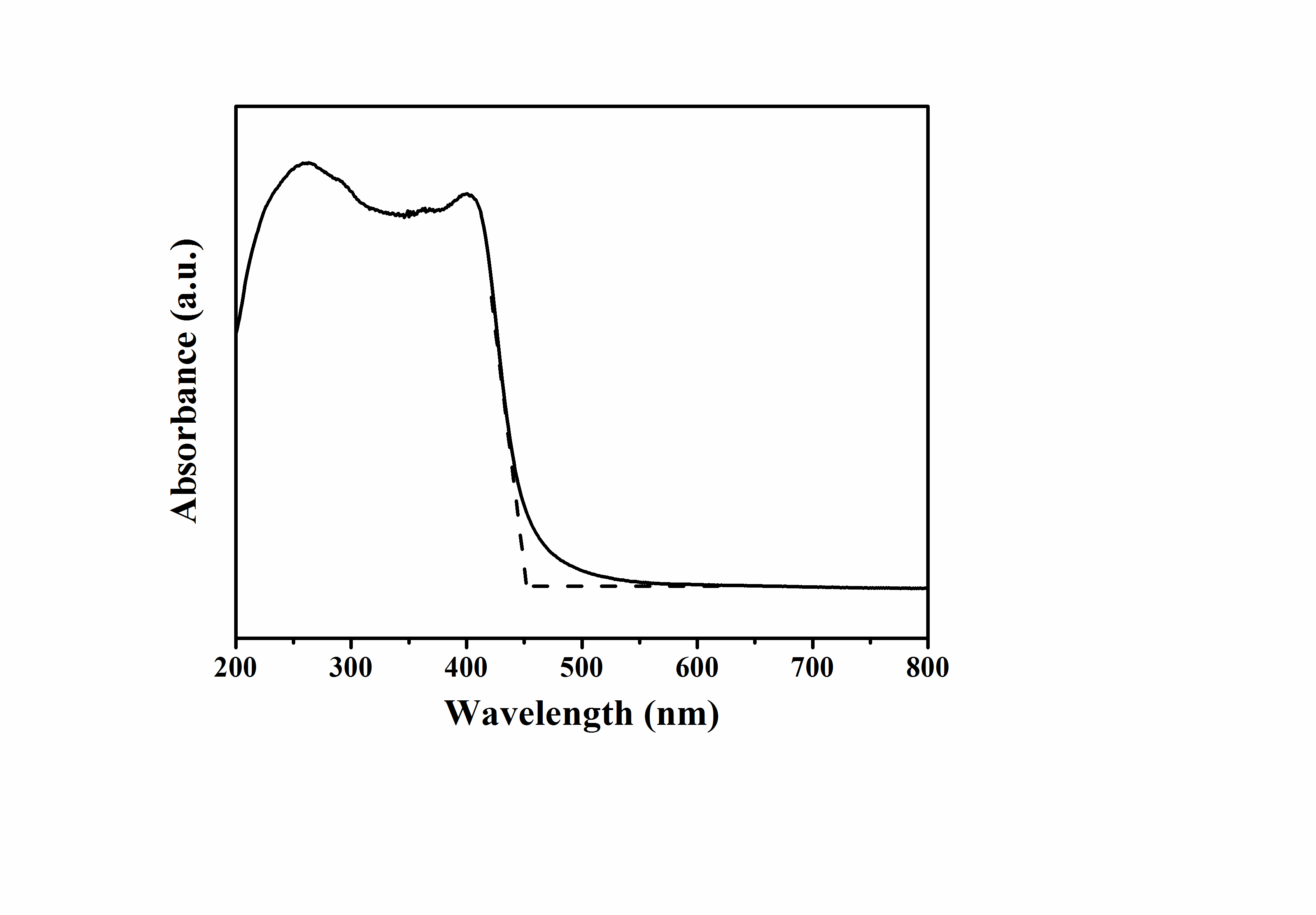


**Figure S5**  Ultraviolet–visible diffuse reﬂectance spectrum of the polymeric Cd_3_(TMT)_2_.


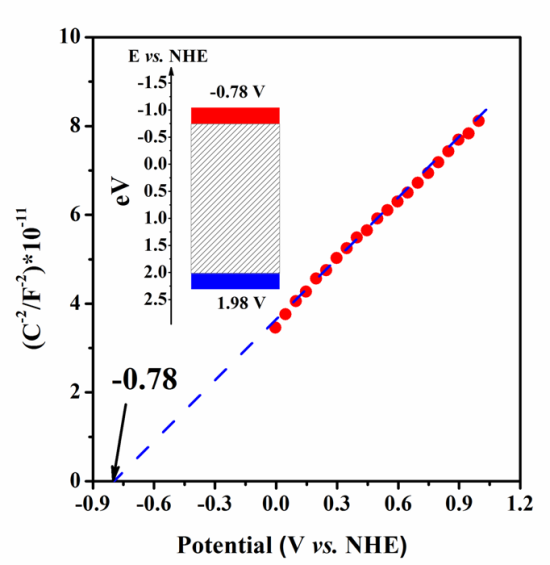

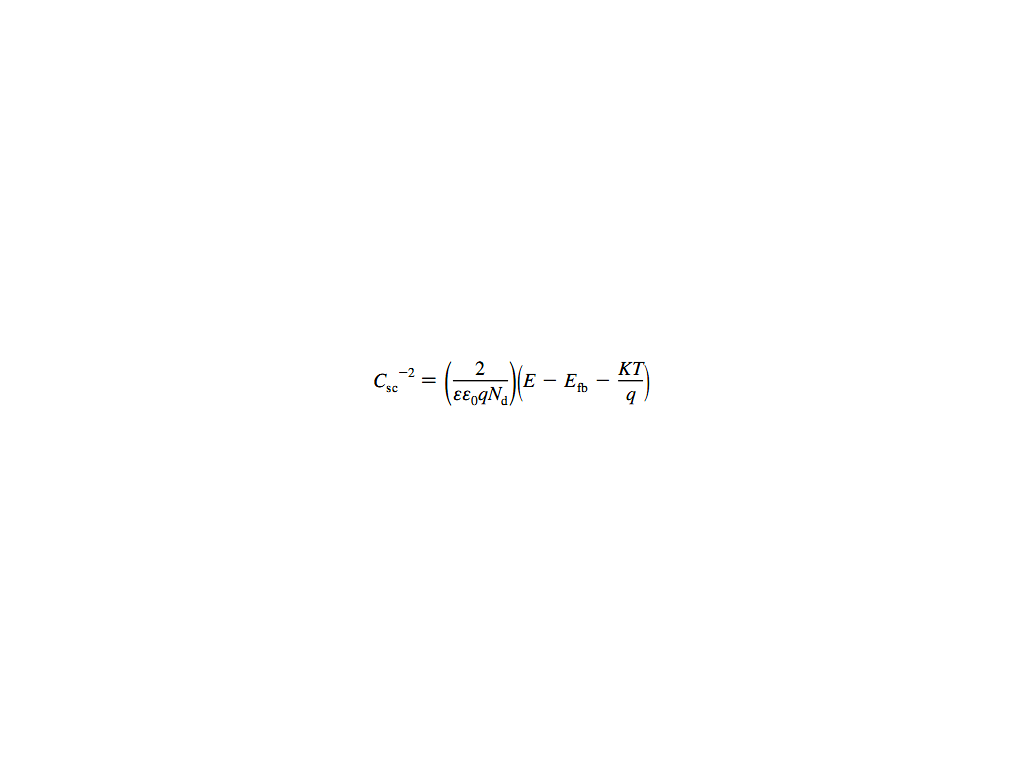


**Figure S6** Mott-Schottky plot of the bare coordination polymer, the inset is the derivated band structure of Cd_3_(TMT)_2_ and the Mott-Schottky equation (where C_sc_ is the total capacitance of the space charge region, E is the potential, E_fb_ is the flat band potential, *K* is Boltzmann’s constant, and *T* is the temperature, ε is dielectric constant of the semiconductor, ε_o_ is permittivity of free space, N is donor density.).


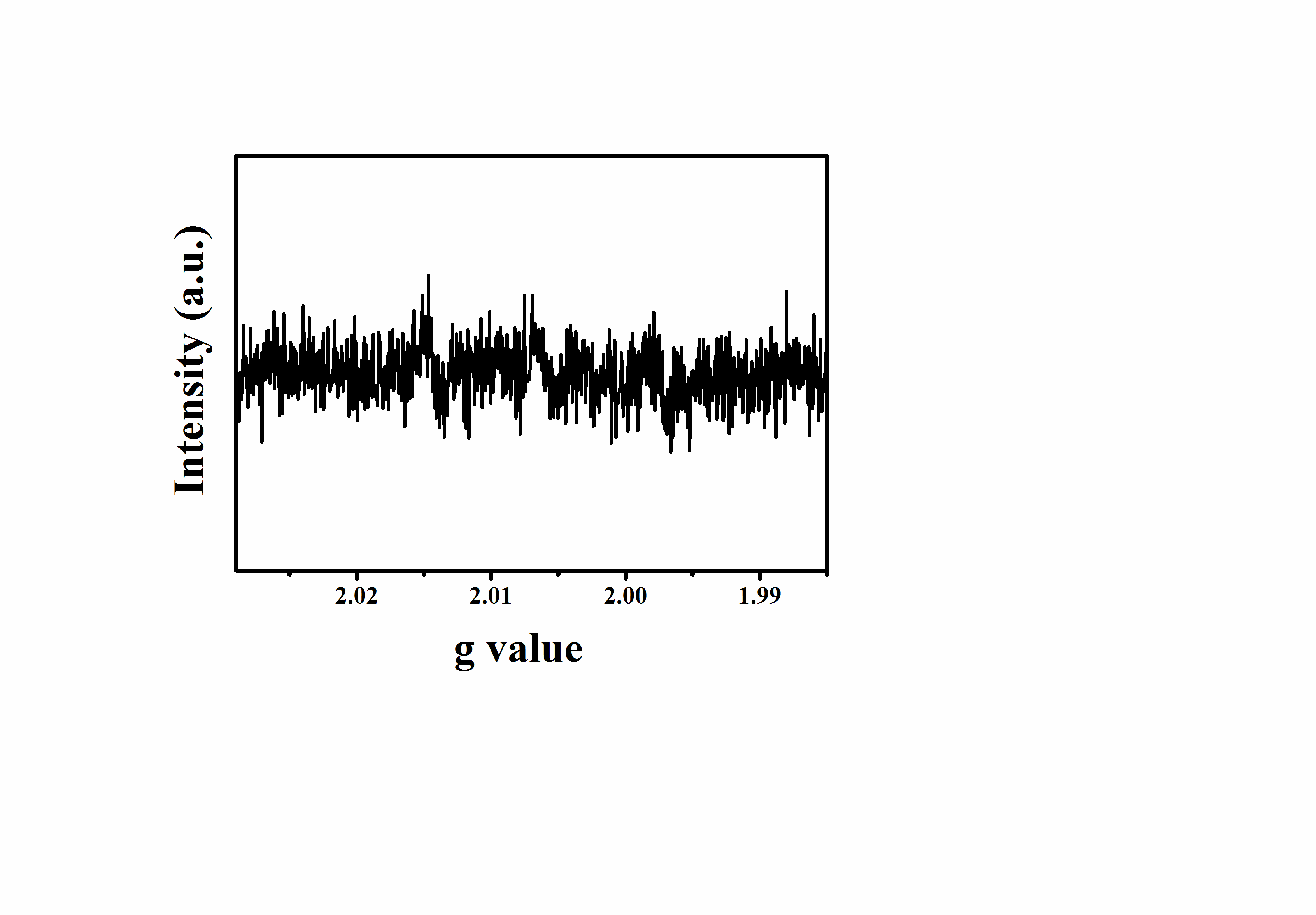


**Figure S7** ESR detection of •OH species.

The generation of •OH radicals was investigated by the ESR technique with DMPO.[^8^](#_ENREF_1)

**
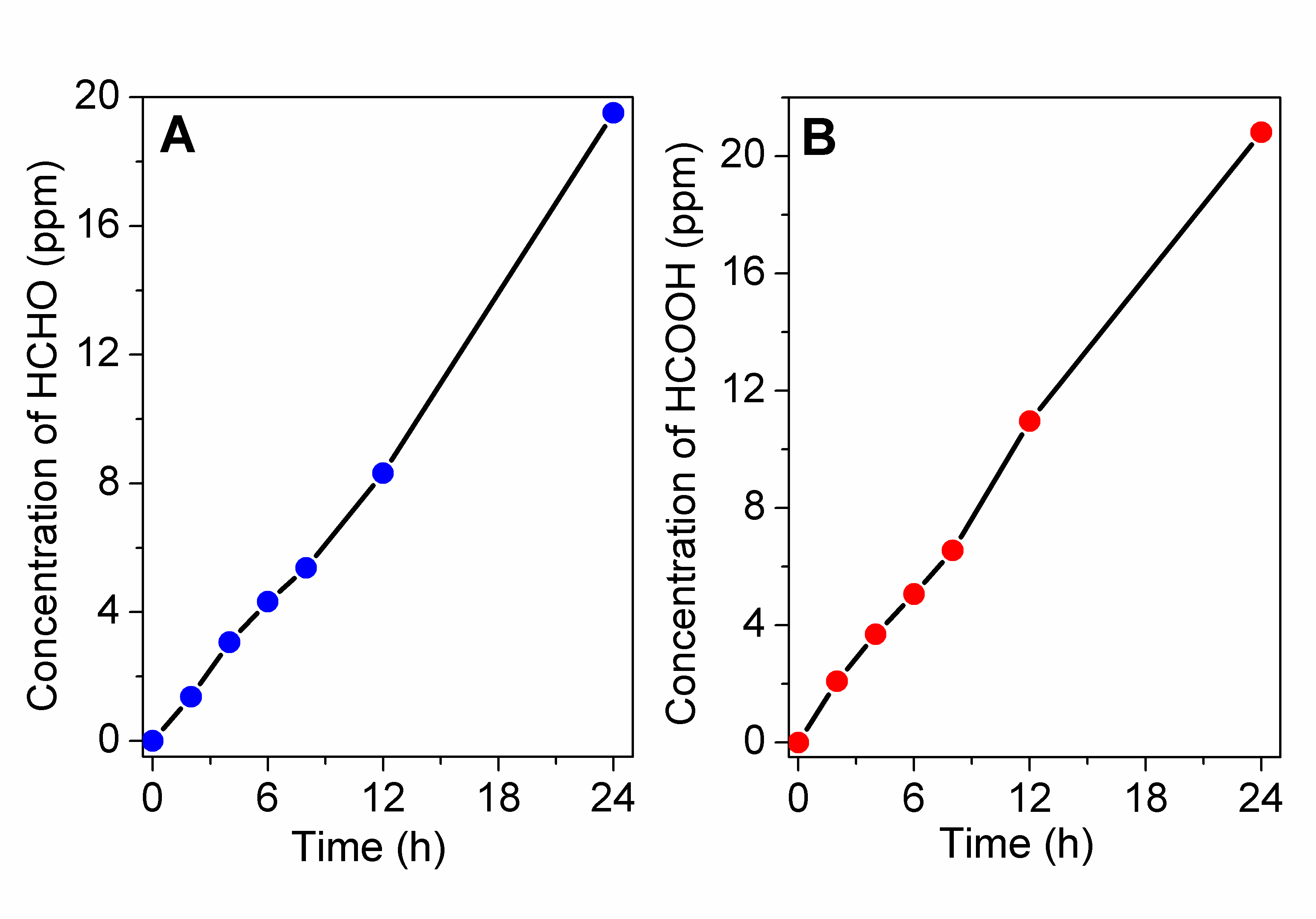
**

**Figure S8** Time courses of the concentration of HCHO (A) and HCOOH (B) over the polymer from methanol aqueous solution. Reaction conditions: 80 mg catalyst dispersed in 19 ml distilled water mixed with 1 ml methanol.


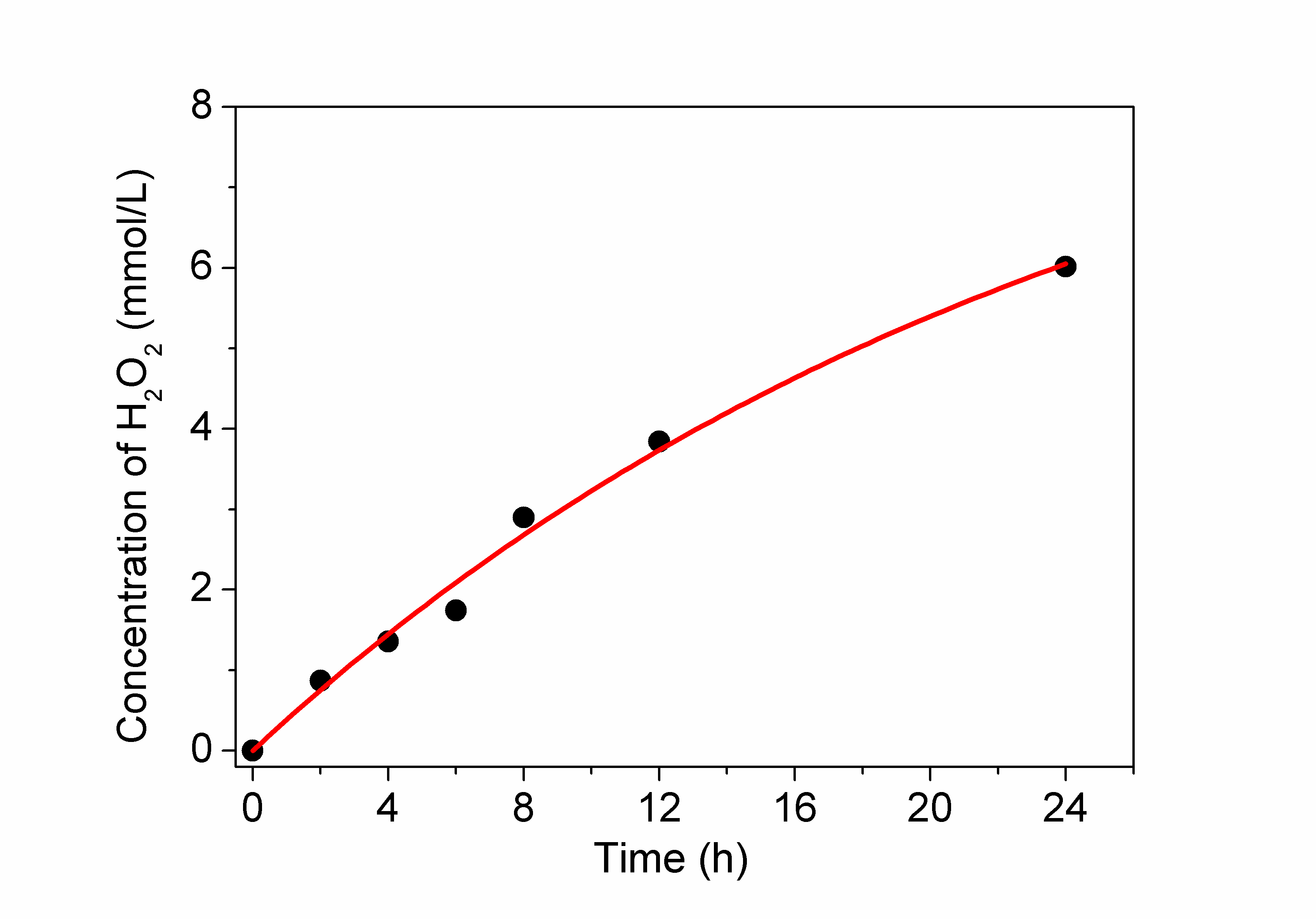


**Figure S9** Time-dependent change in H_2_O_2_ concentration under visible-light irradiation over the polymer from methanol aqueous solution. Reaction conditions: 80 mg catalyst dispersed in 19 ml distilled water mixed with 1 ml methanol.The line is the calculated results using the equation: [H_2_O_2_]= (k_f_/k_d_){1-exp(1-k_d_t)}.


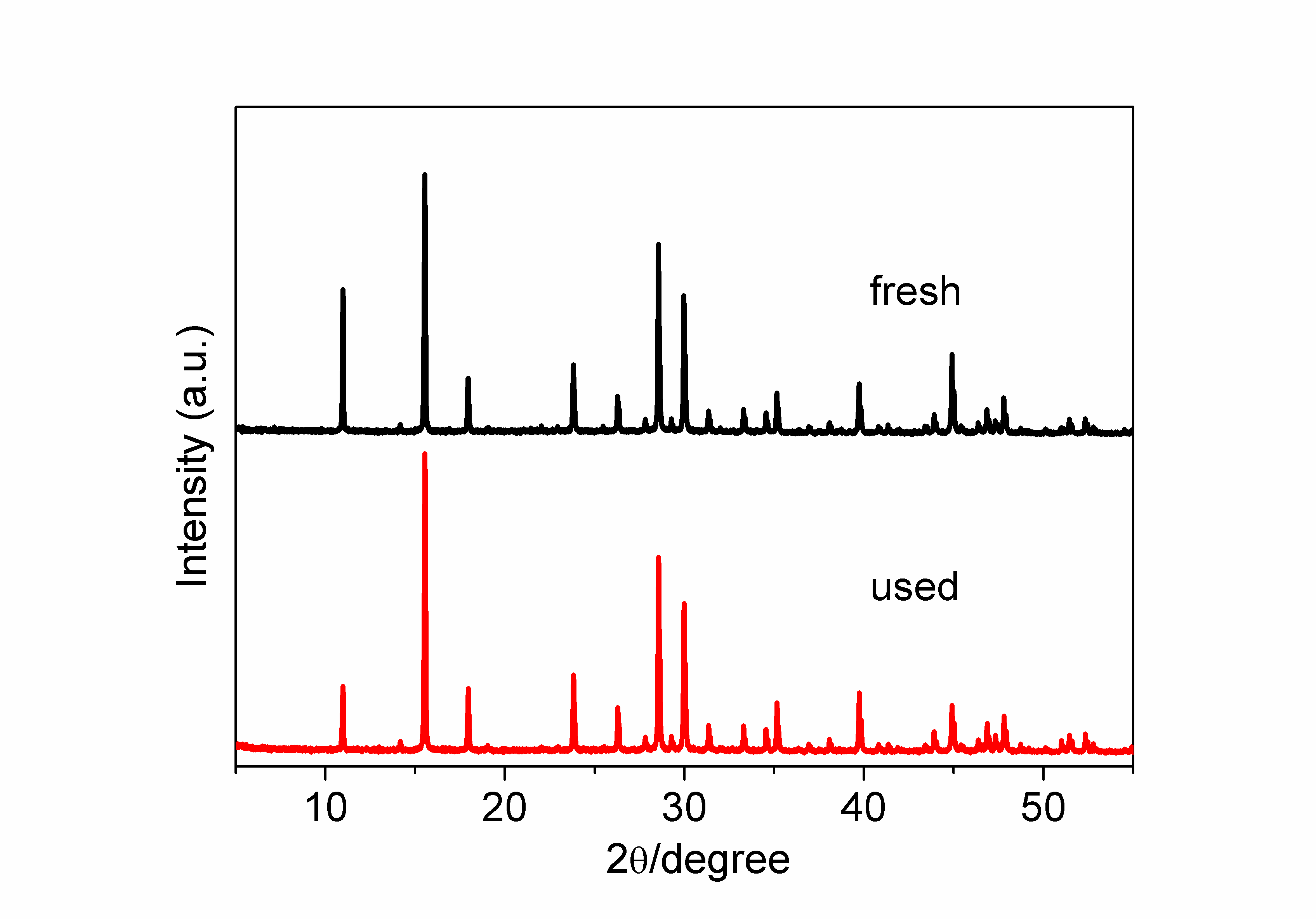


**Figure S10** XRD patterns of Cd_3_(TMT)_2_ polymer before and after photocatalytic reaction


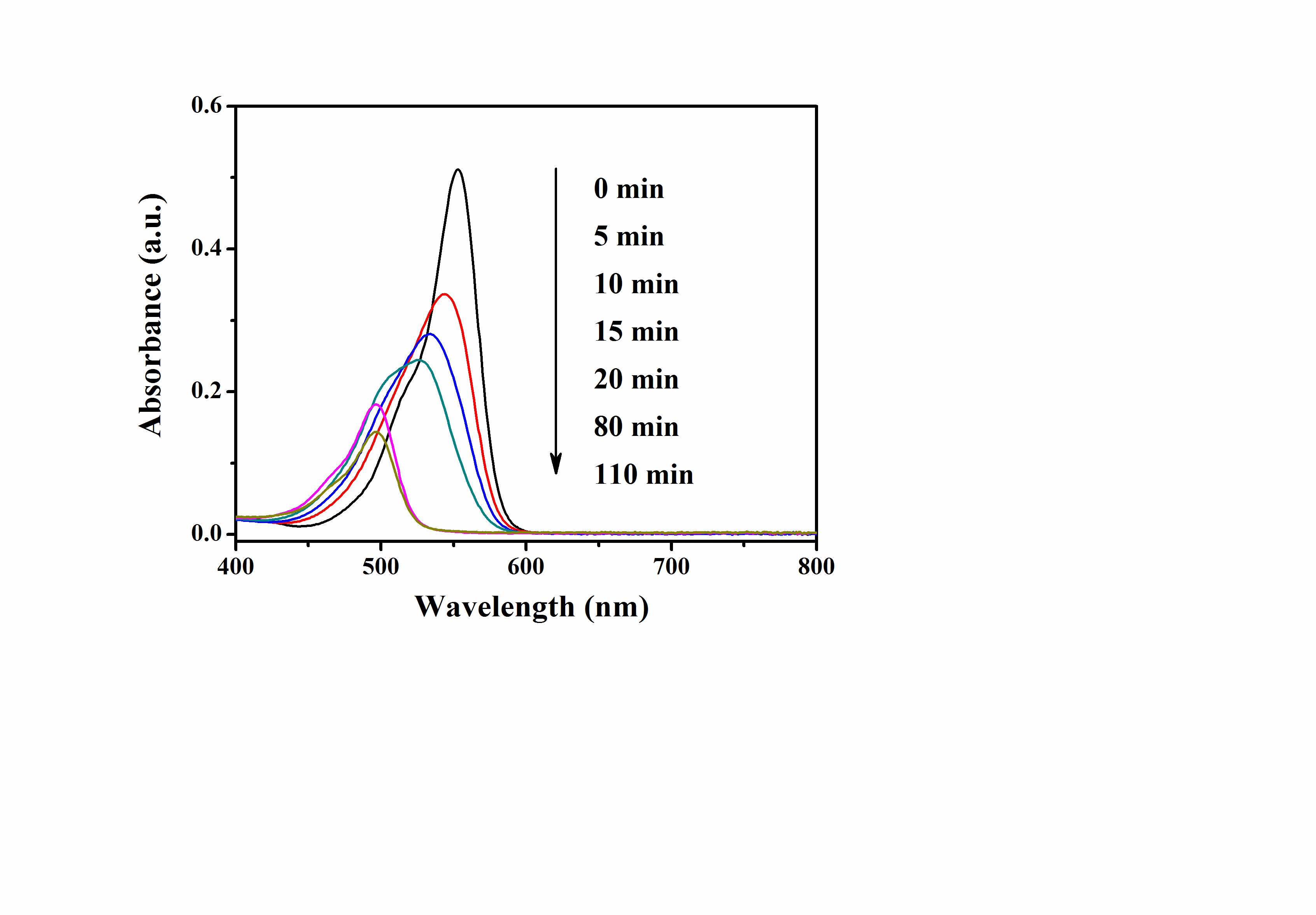


**Figure S11** UV-vis spectral changes of RhB aqueous over the Cd_3_(TMT)_2_ photocatalyst under visible light irradiation (λ > 420nm).

Photocatalytic activity of Cd_3_(TMT)_2_ was evaluated by degradation of RhB under visible light irradiation of an 500 W tungsten halogen lamp with cutoff filter L42 (providing light irradiation of wavelength longer than 420 nm) and a water filter (to prevent IR irradiation). The intensity of the incident light was *ca.* 60 mW/cm^2^. The as-prepared sample (50 mg) was suspended in 80 mL RhB aqueous in a pyrex reactor, the dye concentration was 4.3 mg/L. Before the light was turned on, the suspension was stirred vigorously in the dark for more than 90 min to ensure establishment of an adsorption-desorption equilibrium of dye on the sample surface. At given irradiation time intervals, *ca.* 3.0 mL of the reaction suspension was sampled, and separated by filtration. The filtrates were analyzed by monitoring the maximum absorption variations of RhB on the UV–vis spectrophotometer (Varian Cary 50, USA).


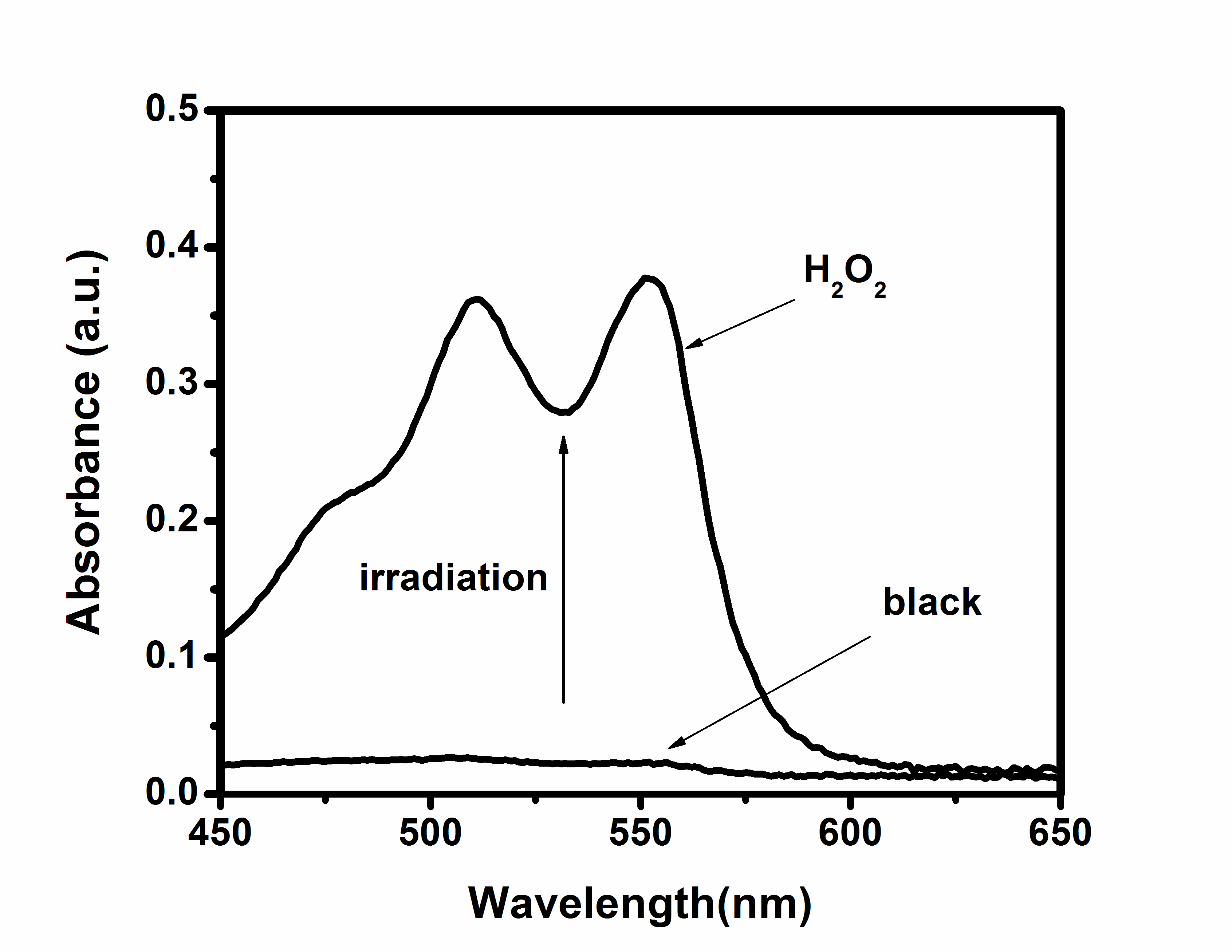


**Figure S12** Detection of H_2_O_2_ using DPD method after photocatalytic degradation of RhB.

The experiment is to determine the formation of H_2_O_2_ after photocatalytic degradation of RhB over Cd_3_(TMT)_2_ under visible light irradiation, based on the formation of H_2_O_2_-DPD-POD adduct that shows commonly two absorption peaks centered at *ca.* 510 and 550 nm.^9^ It appears that after adding DPD and POD in reaction system, two obvious peaks were observed, indicating that H_2_O_2_ was produced after the photocatalytic RhB degradation.

**Supplementary References**

1. Matlock, M. M., Henke, K. R., Atwood, D. A. & Robertson, D. [Aqueous leaching properties and environmental implications of cadmium, lead and zinc Trimercaptotriazine (TMT) compounds](http://www.sciencedirect.com/science/article/pii/S0043135401000914). *Water Res.* **35**, 3649-3655 (2001).

2. Bailey, J. R. et al. Transition metal complexes of 2,4,6-trimercapto-1,3,5-triazine (TMT): potential precursors to nanoparticulate metal sulfides. *J. Organometal. Chem*. 623, 185-190 (2001).

3. Loughran, G. A., Ehlers, G. F. L., Crawford, W. J., Burkett, J. L. & Ray, J. D. The infrared spectra of some new derivatives of s-triazine. *Appl. Spectrosc*. 18, 129-134 (1964).

4. Kucharski, M. & Chmiel-Szukiewicz, E. Reactions of trithiocyanuric acid with oxiranes. I. synthesis of polyetherols. *J. Appl. Polym. Sci*. 76, 439-445 (2000).

5. Haiduc, I., Mahon, M. F., Molloy, K. C. & Venter, M. M. Synthesis and spectral characterisation of organotin(IV) 1,3,5-triazine-2,4,6-trithiolato complexes, including the crystal structures of 1,3,5-(R_3_Sn)_3_C_3_N_3_S_3_ (R=Me, Ph). *J. Organometal. Chem*. 627, 6-12 (2001).

6. Henke, K. R., Bryan, J. C. & Elless, M. P. Structure and powder diffraction pattern of 2,4,6-Trimercapto-s-triazine, trisodium salt (Na_3_S_3_C_3_N_3_•9H_2_O). *Powder Diffraction* 12, 7-12 (1997).

7. Beezer, A. E. & Chudy, J. C. Elucidation of coordination polymer stoichiometry via thermometric titrimetry: Metal complexes of trithiocyanuric acid. *Thermochimica Acta* 6, 231-237 (1973).

8. Chen, C. et al. Photocatalysis by Titanium Dioxide and Polyoxometalate/TiO_2_ Cocatalysts. Intermediates and Mechanistic Study. *Environ. Sci. Techn*. 38, 329-337 (2003).

9. Bader, H., Sturzenegger, V. & Hoigné, J. Photometric method for the determination of low concentrations of hydrogen peroxide by the peroxidase catalyzed oxidation of N,N-diethyl-p-phenylenediamine (DPD). *Water Res*. 22, 1109-1115 (1988).
